# Editorial: Insights in microbial immunology: 2022

**DOI:** 10.3389/fimmu.2023.1219083

**Published:** 2023-05-25

**Authors:** Ian Marriott, Imtiaz A. Khan, Ulrich E. Schaible

**Affiliations:** ^1^ Department of Biological Sciences, University of North Carolina at Charlotte, Charlotte, NC, United States; ^2^ Department of Microbiology, Immunology, and Tropical Medicine, School of Medicine and Health Sciences, The George Washington University, Washington, DC, United States; ^3^ Department of Cellular Microbiology, Research Center Borstel, Leibniz Lung Center, Borstel, Germany; ^4^ Department of Immunochemistry and Biochemical Microbiology, University of Lübeck, Lübeck, Germany

**Keywords:** microbial immunology, achievements and challenges, microbiota, state-of the art review, multi-omic analyses, infection immunology

The third decade of the 21st Century has seen incredible advances in immunology and so Frontiers has organized a series of Research Topics to highlight the latest achievements in this field. This editorial initiative has centered on describing the latest discoveries and future perspectives, while recognizing current challenges in the field. Accordingly, we solicited forward-looking articles that describe state-of-the-art approaches and recent developments in microbial immunology, and that identify the present obstacles and opportunities for further progress. This call resulted in the following collection of eight articles that discuss the tremendous advances that have been made over the last decade in our understanding of the host-pathogen interactions that underlie both disease pathogenesis and the maintenance of good health. This has been made possible, in large part, by the development and use of sophisticated new technologies that have permitted comprehensive analyses of changes in microbial communities and subsequent host responses that underlie disease development, and this is reflected in the following contributions.

The gut microbiota is complex and has vital functions in digestion, metabolism, vitamin supplementation, pathogen resistance, and maintenance of a functional intestinal wall barrier. It also plays a critical role in the development and function of the immune system and so has a considerable impact on human health and disease. As such, understanding the distribution and function of this ecosystem has been a recent research hotspot and there has been considerable interest in diseases that show links between immune responses and alterations in the gut microbiota, which might be exploitable as early diagnosis biomarkers or even as a target for therapeutic intervention. Examples of this include the application of fecal microbiota transplantation to treat refractory *Clostridioides difficile* infections, to overcome resistance to anti-PD-1 therapy in melanoma patients, or to inhibit endogenous insulin decline in type I diabetes patients.

In a bibliometric analysis, Ni et al. have identified recent progress and developing trends in the study of gut microbiota and host immunity. In their studies, global publications in this field from 2011 to 2021 were extracted from the Web of Science and evaluated by bibliometric and visual methods to determine spatial and temporal distributions of study status, identify the major and highly cited scholars, reveal emerging thematic frontiers, and to identify current knowledge gaps. Their analysis has served to highlight that this field remains highly topical and has received widespread attention across the globe, thereby facilitating studies in disparate populations with regard to geographical and dietary differences. The US appears to have made the largest number of contributions to the field, followed by China, and has also led in terms of the overall impact. This work has been communicated in highly regarded journals, most notably *Immunity*, although our own *Frontiers in Immunology* has also been well represented in this regard.

While the most frequently mentioned keyword was, unsurprisingly, COVID, linked to studies investigating changes in microbiome composition associated with elevated inflammatory mediator levels that could impact disease course and patient outcomes, their analysis also shows that the relationship between the gut microbiota and cancer, central nervous system disorders (the gut-brain axis), and autoimmunity, has been under particularly extensive scrutiny. Of important note was the interest in its links to cancer development and immunotherapy efficacy, specifically, the therapeutic potential of modulating the gut microbiome in patients receiving immune checkpoint inhibitors for the treatment of cancer to circumvent resistance. Furthermore, the newest salient keyword was short-chain fatty acid (SCFA) based on the suggested ability of its butyrate and propionate metabolites to induce Treg differentiation, thereby bridging gut microbiota and host immune system regulation.

With regard to challenges to the field, this bibliometric analysis identified the need for improved bacterial strain screening and breakthrough innovations in related experimental techniques, and the significant lag between animal studies and clinically applied research required to support translation was remarked upon. Furthermore, the conflicting results seen in the study of probiotics in even high-quality studies was noted, which suggests that such approaches might require the development of personalized approaches for prophylaxis or therapy.

Relative to the study of the bacterial composition of the microbiome, relatively little work has been done on the gut mycobiota. While it is an almost negligible percentage of the gut microbiome compared to bacteria, the mycobiota has a significant effect on human health and disease. It is essential for the development and maintenance of the human immune system and shows alterations in both intestinal and extraintestinal diseases. In this Research Topic, Zhang et al. comprehensively review research progress in the study of the gut mycobiota, with an emphasis on its role and composition changes in patients with gastrointestinal and non-gastrointestinal neoplasms. Importantly, they describe the role of C-type lectin fungal pattern recognition receptors (PRRs), such as the dectins and macrophage-inducible C-type lectin (Mincle), and their downstream signaling pathways, most notably caspase recruitment domain-containing protein 9 (CARD9), in tumor development and anti-tumor responses. Together, the available evidence suggests that altering the mycobiome could ameliorate disease, and anti-fungal treatment and/or the targeting of such immune components could be attractive therapeutic options for even aggressive cancers, such as pancreatic ductal adenocarcinoma. However, the authors have noted the need for large-scale, long-term, prospective and longitudinal multi-omics studies to determine whether mycobiome changes are causal or consequences in malignant tumors, and to identify new targets for cancer control.

In a mini-review in this collection, Hu et al. expound on recent clinical and basic science advances in our understanding of the mechanisms and function of CARD9. This critical downstream PRR adaptor molecule is abundantly expressed by myeloid cells and is an indispensable regulator in antifungal immunity. As such, much recent research has focused on elucidating the association between CARD9 and susceptibility to fungal infections, and its potential exploitation as a target for therapeutic intervention. The authors discuss the latest CARD9-based treatment and prevention strategies, and current prospects for this line of research. However, they note that species-specific anti-fungal mechanisms appear to exist. For example, a CARD9 independent and Syk dependent mechanism has been identified that seems to have a bigger impact on anti-Candida immunity than those mediated by CARD9.

In addition to its anti-fungal role, CARD9 also mediates PRR-induced immune responses to bacterial, viral, and parasitic infections and this is discussed in this article. Signaling *via* classic C-lectin receptors, such as Dectin-2, participates in antibacterial immunity, and the Mincle/CARD9 axis has been shown to play a role in early anti-viral immunity. However, PRRs can also initiate antibacterial activity *via* a CARD9-independent signaling pathway that interweaves with CARD9-dependent mechanisms to exert a bactericidal effect, and evidence is conflicting as to whether CARD9-mediated responses to viral infection are beneficial or contribute to immune-pathological damage. Such complexities may be attributable to host species, tissue type, or even pathogen species, specific differences. Clearly, more study of the relationship between classic and specific pathways is essential if the development and use of CARD9 inhibitors as therapeutic agents is to advance.

Despite being a low biomass relative to the gut microbiome, the lung harbors a distinct and dynamic community of commensal microorganisms. This lung microbiota interacts bidirectionally with pulmonary immunity and can significantly impact immune responses in health and disease. In a review by Huynh et al., the authors discuss our current understanding of the interactions between the lung microbiota and the immune system in the context of the unique biology of the lung microenvironment, and how they are linked to lung cancer development and outcomes. In this work, the authors frequently have to refer to studies in the gut to underscore the potential role of the microbiota in lung cancers, and lament that research has lagged in this area behind other lung diseases and studies of the gut microbiota. They highlight the remaining knowledge gaps in this field, and note that improved microbial extraction and standardization methods, which increase the granularity of microbial species identification, will be the key to further research and improved patient outcomes. Furthermore, they identify the urgent need to define the effect of radiation therapy or chemotherapy on the lung microbiota and determine how the lung microbiota may impact the efficacy of these common cancer treatments.

While the mechanisms that underlie the development of irreversible pulmonary fibrosis are presently unclear, it is apparent that changes in the lung microbiota affect its progress. In another review article in this collection, Zhang et al. attempt to summarize the available evidence for a relationship between the pulmonary microbiota and immune regulation as it pertains to pulmonary fibrosis. In this piece, they place particular emphasis on the role of core fucosylation, an important functional glycoprotein modification, in pathological immunity. These authors discuss the key role that core fucosylation plays in myofibroblast accumulation and extracellular matrix deposition, and suggest that its modification might be exploited as a new strategy to limit pulmonary fibrosis. However, they describe similar problems in this area of research with regard to sampling methods, and note the need for larger and more rigorous studies.

Regarding new methodologies that promote more robust studies in the field of microbial immunology, perhaps none have been more important than advances in proteomics. These methods have proven instrumental in the identification of disease biomarkers for early diagnosis and the delineation of the mechanisms underlying disease pathology. In this collection, Boada et al. have used unbiased discovery-based serum proteomics to examine the serum proteomes of study participants with two common arthropod-borne infections, Lyme disease that is caused by the spirochete *Borrelia burgdorferi*, and infection with the flavivirus West Nile virus. Two methods, a depletion-based high throughput shotgun proteomics pipeline and a non-depleting blotting-based low-throughput platform proteomics method, MStern blotting, were employed to study the LC/MS based serum proteome of samples collected at the time of diagnosis and during convalescence. These parallel approaches identified both common and differentially abundant proteins in the acute and recovery phases of each infection, and also some that distinguished localized from systemic, and symptomatic from asymptomatic, infections. Interestingly, these complementary profiling techniques revealed many similarities in the serum proteome responses despite the disparate nature of the pathogens, especially products involved in inflammation and in innate immune regulation and tissue repair. Furthermore, significant changes in the proteome were observed for several months after infection despite clinical disease resolution. As such, these initial exploratory findings may point to common pathways and generalizable targets for disease modifying interventions.

In an example of how defining the mechanisms underlying pathology can identify targets for therapeutic intervention, Katharina Kubatzky reviews the role of *Pasteurella multocida* toxin (PMT) in infections caused by this Gram-negative bacterium. *P. multocida* causes disease in domestic and wildlife animals, and toxigenic strains are commonly found in pigs where PMT mediates the destruction of nasal turbinate bones. In this article, the author describes how PMT targets heterotrimeric G proteins to promote the generation and/or activation of bone resorbing osteoclasts, while simultaneously inhibiting bone forming osteoblast activity, which leads to bone loss. The impact of immunity and pathogenicity factors on disease severity and outcome are outlined, and the evidence for PMT-mediated effects on other organs is also summarized. Given the mechanism of action of PMT, it is suggested that this toxin might be exploited as a biotechnological tool for the study of human pathologies such as cancer and autoimmunity.

Finally, inhibitors that block complement activation pathways have been introduced clinically but have significant safety concerns due to elevated risks of infection by encapsulated bacteria. As such, inhibitors of specific complement components are currently under investigation as alternative treatment options to limit these risks. Defense against *Haemophilus influenzae*, a major cause of invasive bacterial infection in children worldwide, is dependent on antibody and complement-mediated immunity, and the introduction of a capsular polysaccharide-protein conjugate vaccine has virtually eliminated such infections where employed. Despite this, some vaccinated individuals remain susceptible to *H. influenzae* infection. In original research in this collection, Muri et al. evaluated the impact of capsule-specific antibodies and inhibitors of C3, C5, and the alternative and the lectin complement pathways, on *H. influenzae* killing by serum or reconstituted blood from individuals both before and after vaccination. Their observations indicate that serum membrane attack complex-mediated bactericidal activity is more efficient than blood cell opsonophagocytosis in host defense against *H. influenzae* type B. Importantly, the finding that alternative pathway inhibitors have less of an effect on this defense over C3 and C5 inhibitors, especially after vaccination, support the use of these therapeutic agents in patients with increased susceptibility to *H. influenzae* infection. Clearly, these data set the stage for future clinical studies to confirm the improved safety profile of such agents.

Together, this Research Topic provides examples of the extraordinary progress made in the field of microbial immunology over the last decade and highlights the leading role played by the latest advances, particularly in multi-omics technologies, in these advances. These will likely set the stage for new disease prevention approaches and improved diagnosis and treatment options. Moreover, this Research Topic also highlights the challenges that remain in this area, most notably the need for improved sampling methodologies and the significant lag that continues to be apparent between animal studies and clinically applied research.

## Author contributions

IM wrote this editorial and IK and US edited and approved it.

